# Acute effects of predominantly inertial load-induced post-activation potentiation on upper-body muscle mechanics: implications for racket sports performance

**DOI:** 10.3389/fspor.2026.1732933

**Published:** 2026-02-24

**Authors:** Sonja Kocić Pajić, Aleksandar Nedeljković, Goran Janković, David Nikolić, Marko Ćosić, Filip Kukić

**Affiliations:** 1 Faculty of Sport and Physical Education, University of Belgrade, Belgrade, Serbia; 2 School of Athletic Performance, Shanghai University of Sport, Shanghai, China; 3 Faculty of Physical Education and Sports, University of Banja Luka, Banja Luka, RS, Bosnia and Herzegovina

**Keywords:** force-velocity relationship, movement velocity, power improvement, racket sports, resistance training

## Abstract

**Introduction:**

This study investigated the acute effects of post-activation potentiation (PAP) induced by predominantly inertial loading on upper-body muscle mechanics, with particular consideration for applications in racket sports performance.

**Methods:**

Fifteen participants experienced in resistance training completed two testing sessions. During the first session, participants underwent anthropometric assessment, familiarization with the bench press throw under inertial resistance, and determination of one-repetition maximum (1RM). The second session included a pre-test, a PAP protocol consisting of 2 sets of 3 ballistic chest press repetitions at approximately 80%–90% 1RM with inertial loading (rubber bands counteracted gravitational force to provide predominantly inertial load), followed by a post-test after five minutes of standardized rest. Performance variables—movement velocity, power, and maximal force—were recorded.

**Results:**

Results demonstrated significant increases in movement velocity and maximal power output following PAP (*p* ≤ 0.05), while maximal force significantly decreased. Consequently, the force–velocity relationship exhibited a rightward shift.

**Conclusion:**

These findings indicate that predominantly inertial loading can effectively elicit PAP and enhance explosive upper-body performance. Such effects may be particularly beneficial for athletes in racket sports, where rapid, high-velocity upper-limb actions performed under light external loads are critical for optimizing stroke power and precision.

## 1 Introduction

Numerous sports require athletes to generate high levels of power to perform successfully. While the development of lower-limb power has been extensively researched (1), upper-limb power generation remains less explored. The upper limbs typically operate within open kinetic chains and involve movements performed against relatively light loads, whereas the lower limbs often function in closed kinetic chains and require greater resistance (1). For example, the legs propel the body through space (e.g., change-of-direction ability), while the arms generate and transfer force to external objects (e.g., swing the racquet and hit the ball). In racket sports such as tennis, badminton, table tennis, baseball, and squash the ability to generate explosive upper-limb movements under relatively light external loads is a key determinant of performance. Stroke efficiency and shot speed rely heavily on the athlete's capacity to produce high movement velocities while maintaining control and precision. This combination of speed-strength demands makes racket sports an ideal context for studying upper-body post-activation potentiation (PAP), particularly when induced by inertial loading. Understanding how inertial PAP influences velocity and power in upper-limb movements can therefore provide valuable insights for optimizing stroke mechanics and on-court performance in these sports.

Post-activation potentiation (PAP) is a physiological phenomenon characterized by a transient enhancement in muscle performance following a high-intensity conditioning activity (2, 3). This effect occurs due to increased phosphorylation of myosin regulatory light chains, heightened motor unit recruitment, and enhanced neuromuscular efficiency (4, 5), which collectively improve force production and rate of force development. PAP is typically achieved through the execution of heavy resistance exercises, ballistic movements, or isometric contractions, performed prior to explosive tasks (3, 6). The effectiveness of PAP depends on various factors, including the type, intensity, and volume of the conditioning activity, as well as the recovery period between the conditioning stimulus and subsequent performance (3, 6).

The intensity of the conditioning activity plays a crucial role in determining the magnitude and duration of the PAP effect. Research indicates that heavier loads, typically around 80%–90% of one-repetition maximum (1RM), are more effective in eliciting PAP compared to lighter loads. Seitz and Haff (7) demonstrated that higher-intensity resistance exercises produced greater improvements in power output, particularly in well-trained individuals. Similarly, a study by Tillin and Bishop (8) found that maximal and near-maximal contractions led to significant short-term enhancements in explosive performance due to increased motor unit activation and myosin light chain phosphorylation. However, excessive intensity may induce fatigue, potentially diminishing PAP benefits. Esformes et al. (9) reported that submaximal loads (60%–85% 1RM) struck a balance between eliciting PAP and minimizing fatigue, optimizing subsequent performance. Additionally, studies highlighted those characteristics, such as training experience and recovery capacity, influence the optimal intensity required to maximize PAP effects (4, 7).

The type of conditioning activity significantly influences the magnitude of the PAP effect, with squat-based protocols being the most studied, while some studies have also explored the bench press and plyometric training. For example, Seitz and Haff (7) demonstrated that performing back squats at 85%–90% 1RM significantly enhanced subsequent sprint performance, highlighting the effectiveness of heavy lower-body resistance exercises in eliciting PAP. Similarly, Houlton et al. (10) found that 3RM back squats improved counter movement jump performance, suggesting that maximal strength exercises effectively enhance explosive power. In upper-body conditioning, Esformes et al. (9) found that multiple sets of heavy bench press (85% 1RM) improved power output in subsequent explosive push-ups, while Tsoukos et al. (11) demonstrated that performing bench press at moderate loads (≈60% 1RM) with controlled velocity loss (10%) acutely enhanced bench press throw performance by 7%–9%, showing that submaximal, velocity-based protocols can optimize potentiation while minimizing fatigue. Furthermore, Maloney et al. (12) observed that plyometric push-ups induced significant improvements in upper-body power, indicating that explosive bodyweight exercises can also serve as effective conditioning activities.

Recent studies have highlighted the importance of load type in shaping the mechanical properties of muscle performance and the interpretation of force-velocity (F–v) profiles. Depending on whether the load is gravitational (W), inertial (I), or combined (W + I), the resulting F–v parameters—maximal force (F₀), velocity (V₀), and power (Pmax)—can vary significantly. For instance, Janković et al. (13) found that inertial loads produce higher peak velocities, gravitational loads favor greater force, and combined loads yield the highest power output. Similarly, Cosic et al. (14) confirmed the linearity of the F–v relationship across load types, with I loads maximizing V₀, W loads favoring F₀, and W + I loads enhancing Pmax. Đurić et al. (15) further demonstrated that long-term training with different load types leads to specific improvements in either force, velocity, or power, depending on the resistance applied. These findings underline the potential of load type to influence neuromuscular responses and PAP outcomes. Therefore, examining inertial loading in upper-body ballistic movements, such as the bench press throw, presents a promising method for targeting distinct performance adaptations through tailored loading strategies.

While PAP effects have been extensively studied in traditional resistance training (weights and inertia) and plyometric training, the effects of inertial loading, specifically using weight plates whose weight was compensated by a constant external force pulling upward, remain unexplored. Furthermore, this type of potentiation has not been explored in upper limbs. Potentiation that enhances power through increased movement velocity (i.e., speed-strength) is highly relevant for sports in which success depends on rapid force production and precise timing under light-load conditions. This neuromuscular mechanism is particularly relevant in racket sports, where rapid upper-limb actions such as serves, smashes, and forehands require explosive, yet precisely timed movements performed with light implements. Understanding how inertial loading can acutely enhance velocity and power provides valuable insights for optimizing performance in tennis, badminton, and similar disciplines. Therefore, the aim of this study was to investigate the effects of potentiation activity using predominantly inertial load on bench press velocity, force, and power as well as on shift in force-velocity profile. Three hypotheses were proposed: (1) PAP with predominantly inertial loading will lead to a significant improvement in velocity and power, (2) PAP with predominantly inertial loading will not result in significant changes in force, and (3) PAP with predominantly inertial loading will lead to a rightward shift in force-velocity relationship.

## 2 Materials and methods

### 2.1 Participants

The study was conducted on a sample of 15 male participants (mean age = 22.5 ± 4.1 yrs, body height = 183.1 ± 5.5 cm, body mass = 86.5 ± 9.2 kg, BMI = 25.8 ± 1.6 kg/m^2^, and percent of body fat = 14.4 ± 3.7%) students of the Faculty of Sport and Physical Education. Sample size estimation was performed using G*Power software, which determined that an optimal sample size for the given study conditions was 12 participants. Participants were included in the study based on the following criteria: a minimum of two years of resistance training experience, engagement in at least six hours of moderate-to-high intensity physical activity per week, and absence of musculoskeletal disorders or recent injuries. To ensure homogeneity of the sample, only participants with a one-repetition maximum (1RM) in the range of 90–100 kg was included. Professional athletes were excluded from the study. This selection criterion aligned with previous studies to facilitate result comparison (14). Additionally, this specific load range was chosen to enable appropriate inertial loading stimulation while considering the mechanical limitations of elastic bands on a specially designed Smith machine. Throughout the study, participants refrained from upper-body resistance training. Prior to participation, all subjects were thoroughly informed about the study's objectives and procedures. Participants were advised to consume a light breakfast with an adequate interval before testing and to arrive well-rested state. Written informed consent was obtained in compliance with ethical guidelines. The study protocol adhered to the principles outlined in the Helsinki Declaration (16) and was approved (no. 02-2324/24-2) by the Ethics Committee of the Faculty of Sport and Physical Education, University of Belgrade, Serbia.

### 2.2 Study design

A repeated-measures study design was employed to determine the effect of inertial loading on the post-activation potentiation (PAP) effect of a ballistic chest press throw, performed on a specially designed Smith machine (14). The Smith machine used in this study allowed only vertical displacement of the barbell, while horizontal and lateral movements were mechanically constrained by the guide rails to ensure a consistent and safe trajectory during ballistic execution. Force, velocity, and power (within-within design) were assessed to investigate whether acute inertial load would produce improvements in power on account of velocity (leftward shift of force-velocity relationship). All measurements were conducted in the morning hours (09:00–11:00 am) at the Methodological-Research Laboratory—Slobodan Jaric of the Faculty of Sport and Physical Education, University of Belgrade. Participants adhered to the following procedural guidelines: wore appropriate sportswear and indoor training shoes.

The study was organized in two sessions. In the first session, testing for each participant included a protocol for anthropometric measurements and familiarization (i.e., introducing the ballistic chest press throw with inertial loading to minimize potential learning effects since none of the participants had prior experience with this type of loading). The grip width on the barbell was determined individually and marked using color markers to ensure a consistent grip width across all tests. The range of motion (ROM) was kept constant across all ballistic trials by maintaining the same starting position and individually marked grip width for each participant. The one-repetition maximum (1RM) was assessed using a standard incremental loading procedure (17). Load intensity for the applied treatment was determined as the percentage of 1RM. Based on the previously estimated 1RM, three different load magnitudes—20%, 50%, and 80% of 1RM—were used to construct the force-velocity relationship, from which three key parameters representing maximal force, velocity, and power were estimated (18). In the second session, participants performed pretest, application of PAP treatment, and posttest after a 5-min rest that showed to be an optimal resting period (7). The PAP treatment involved 2 sets of 3 ballistic chest press repetitions using a load of 80 kg, which, depending on the individual 1RM values of the participants, represented approximately 80%–90% of their maximum, yielding an average relative intensity of 85% 1RM across the sample. Participants were instructed to perform both eccentric and concentric phase of chest press as fast as possible; therefore, contraction tempo (eccentric, amortization, and concentric phases) was self-selected to allow a natural movement pattern that optimized performance. The throws in each series were executed consecutively, one after another, without a pause, in a ballistic manner. The rest period between the series was set at 3 min. Throughout the testing protocol, the subjects were verbally instructed and encouraged to perform the inertial load chest throws as high and as quickly as possible.

### 2.3 Simulation of inertial load

**Figure 1:**
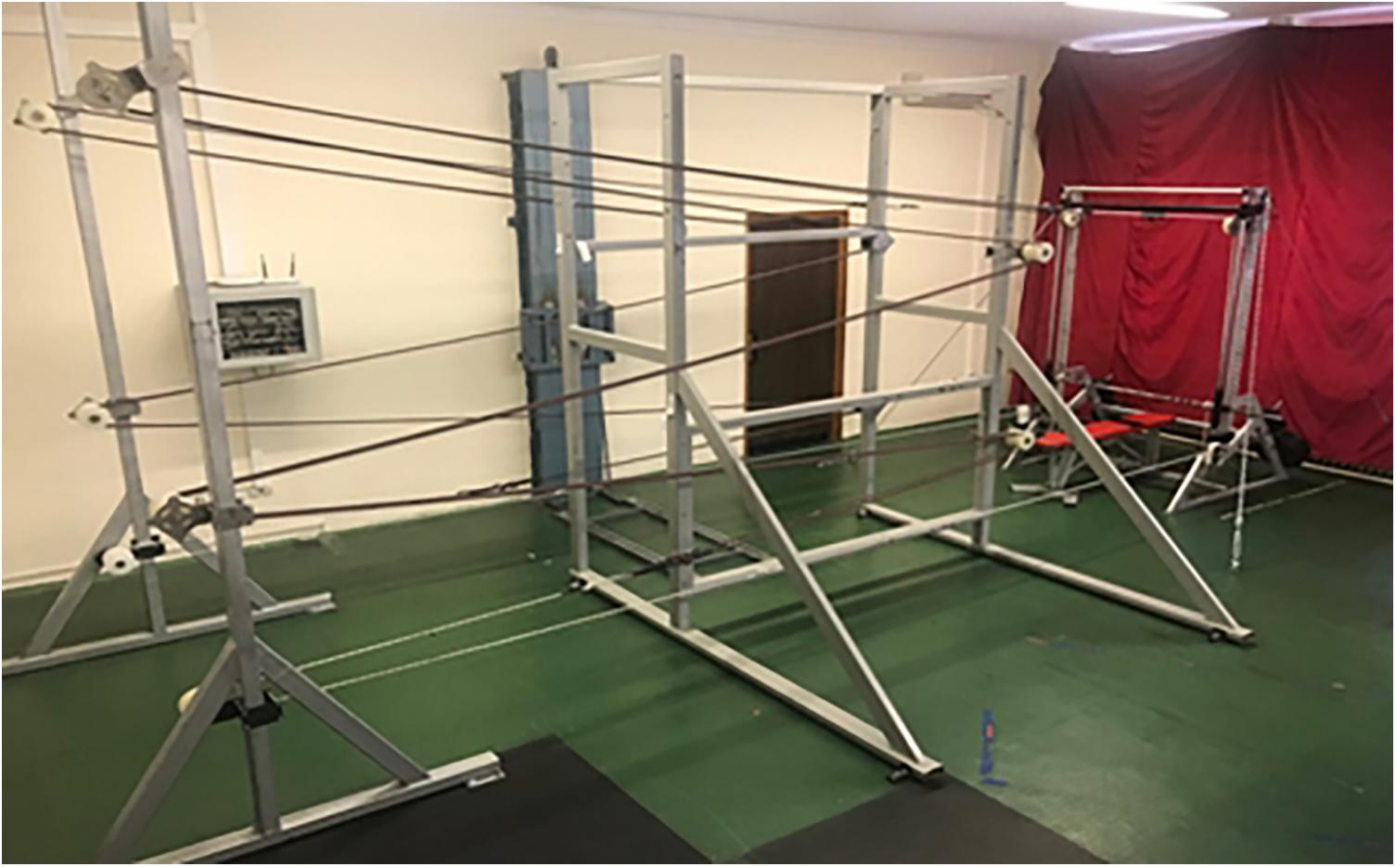
Custom-built Smith machine setup for simulating inertial loading. Elastic bands partially counteracted gravity, allowing ballistic chest press throws to be performed under predominantly inertial conditions with a consistent movement trajectory.

The testing protocol was preceded by an appropriate full-body warm-up and additional 3–4 min of upper body calisthenics. The treatment was performed using a custom-built metal structure equipped with six elastic bands (Tigar a.d., Serbia, 14 mm diameter). The tension of each elastic band was verified prior to testing using a manual calibration procedure with a force gauge (≈98 N) and was maintained nearly constant across the range of motion due to the fixed total system length. Each 14 meters long elastic band was stretched over plastic pulleys in a zigzag formation and connected to the barbell ends with non-elastic ropes (Figure 1). The machine was designed to simulate constant additional force, ensuring that the total length of the stretched system—elastic bands and non-elastic rope—remained constant throughout the movement. This configuration generated an additional load approximating 10 kg per band when fully stretched (six bands × 10 kg = 60 kg). For the purpose of this study, rubber bands were used to counteract the gravitational force induced by the additional weight plates, thereby providing predominantly inertial load. The reference load, consisting of the barbell and upper-body segments, combined both gravity and inertia since it was not affected by the elastic rubber bands. The total load used in the treatment to induce post-activation potentiation effects amounted to 80 kg—of which 20 kg included both gravitational and inertial components, while the remaining 60 kg represented purely inertial resistance. Although approximately 25% of the total external load (≈20 kg) remained under gravitational influence, this portion was necessary to stabilize the movement path. Therefore, while the load was not purely inertial, the muscles were predominantly exposed to acceleration-dependent resistance, representing a valid inertial loading condition during ballistic execution.

The subject began the treatment in a supine position on the Smith machine. With fully extended arms, they established a grip at a pre-marked location on the barbell, while two assistants held the barbell to ensure the subject was unloaded. Once ready for the exercise, the assistants released the barbell with the load, and the subject performed three rapid ballistic barbell throws in an eccentric-concentric mode consecutively. During the final throw, the assistants caught the barbell with the weights. The resistance band system was adjusted to generate force in the opposite direction of gravitational force (the stretched bands were connected to the barbell from above via a non-elastic rope, pulling it upwards), thereby canceling out the mass of the system. As a result, only the system's acceleration—its inertia—remained in effect.

### 2.4 1RM assessment

Before 1RM assessment, participants completed a warm-up, cycling for 3 min, followed by up to 5 min of mobility exercises. They began the treatment in a supine position on the Smith machine, gripping a pre-marked location on the barbell while two assistants held it to ensure unloading. The one-repetition maximum (1RM) assessment was conducted only during the first session. Subjects lay on a bench with bent legs and feet resting on the surface to prevent lower back elevation. They executed the bench press as forcefully and quickly as possible, with only the concentric contraction monitored. The barbell, weighing 20 kg, was gripped 1 cm above the chest with elbows at approximately 90°. Angles were measured with a dual-arm protractor goniometer (Protractor Goniometer, 12in; Prestige Medical, Northridge, CA, USA). Proper form was ensured, preventing bouncing or back arching, with the pelvis and feet in contact with the bench. The actual 1RM was determined within two to three trials, increasing the weight in 2.5 kg increments until failure. The highest successfully lifted load was recorded as the final result, with a minimum 4-minute rest between trials to eliminate fatigue.

### 2.5 Force-velocity profile

In order to determine the individual force-velocity profile, three distinct external loads—corresponding to 20%, 50%, and 80% of each participant's 1RM—were applied during the bench press throw. These loads were chosen solely to enable the construction of a linear regression model representing the force-velocity (F–v) relationship, in accordance with the approach described by Jaric (19), as well as with recent methodological suggestions recommending a three-point approach during initial testing to ensure a more robust identification of the force–velocity intercepts (20). For each load, force and velocity values were captured using the Qualisys motion analysis system. Linear regression analysis was then used to estimate the theoretical maximal isometric force (F₀) as the intercept on the force (y) axis, and the theoretical maximal unloaded velocity (V₀) as the intercept on the velocity (x) axis. From these two parameters, the theoretical maximal power output (Pmax) was calculated using the standard formula Pmax = (F₀ × V₀)/4. The same testing protocol was implemented during both the pretest and posttest phases, enabling the evaluation of acute changes in the force-velocity profile induced by the inertial loading intervention designed to elicit post-activation potentiation.

### 2.6 Statistics

All data were entered into an Excel file and then prepared for the statistical analysis. The statistical analyses were performed using JASP (ver. 0.19.02, University of Amsterdam). Descriptive statistics were calculated as mean and standard deviation. The normality of distribution was tested using Shapiro–Wilk test. Because all variables were normally distributed, repeated measure ANOVA was used to test the treatment effects. Specifically, since the three load levels (20%, 50%, and 80% 1RM) served as input points for deriving individual force–velocity regressions rather than as independent experimental conditions, a one-way repeated measures ANOVA was used to test pre–post differences in derived F–V parameters (F₀, V₀, Pmax), consistent with prior methodological recommendations (13, 14, 19). Force-velocity relationship for pre-test and post-test was determined using the Pearson's correlation coefficient. The significance level was set at *p* < 0.05. The effect sizes were calculated using Cohen's d (21). For the pre-to-post-test differences effect sizes were interpreted as follows: <0.2 (trivial), 0.2–0.5 (small), 0.5–0.8 (moderate), 0.8–1.2 (large), and ≥1.3 (very large). For correlation coefficient, effect sizes were interpreted as <0.2 (trivial), 0.2–0.5 (small), 0.5–0.8 (moderate), and >0.8 (large).

## 3 Results

Descriptive statistics and repeated measure ANOVA *post hoc* are shown in Table 1. The applied treatment had a significant effect on Velocity (F = 11.92, *p* < .001) and Pmax (F = 6.70, *p* < .02), whereby both variables improved. In contrast, a significant (F = 6.52, *p* < .02) decline occurred in Fmax.

**Table 1:** Descriptive statistics and *post hoc* test results.

Variables	Pre-test	Post-test	Paired sample *t*-test		Effect sizes
	Mean (SD)	Mean (SD)	*t*	*p*	
	Min—Max	Min—Max			
Velocity (m/s)	2.60 (0.45)	2.86 (0.37)	−3.45	<.001	−0.62
	1.90–3.32	2.30–3.41			
Fmax (N)	944.97 (66.66)	911.4 (54.65)	2.55	.02	−0.55
	855.96–1,079.55	827.21–1,013.65			
Pmax (W)	613.69 (104.42)	650.09 (82.74)	−2.59	.02	−0.39
	452.95–777.52	499.97–781.82			

**Figure 2:**
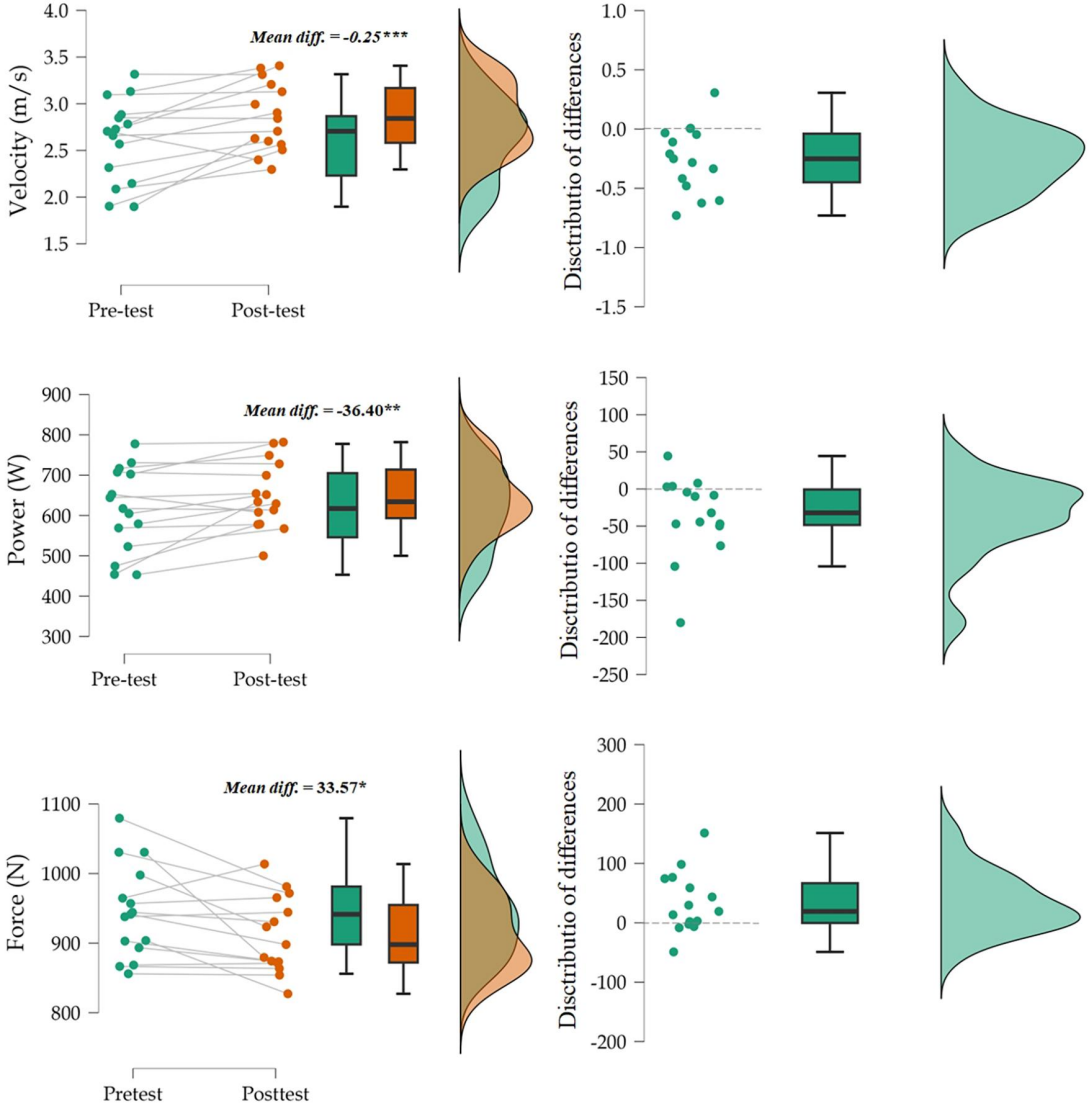
Individual and group changes in velocity, power, and force from pre- to post-test. The narrower post-test distributions indicate reduced inter-individual variability and consistent potentiation effects, with significant increases in velocity and power but a slight decline in maximal force.

As shown in Figure 2, individual and group-level responses demonstrated a consistent increase in velocity and power, accompanied by a modest reduction in maximal force. The relative improvement was larger in velocity (9.6%) than in power (5.9%). Both distributions narrowed at post-test compared to pre-test. The narrower post-test distributions indicate reduced inter-individual variability and suggest that inertial loading elicited a uniform potentiation response across participants. The distribution of differences showed that only one subject performed slower and one remained the same at post-test. Considering power, four subjects performed lower at post-test compared to pre-test. In addition, Fmax reduced significantly and with moderate effect size, although the mean reduction of 33 N (3.4 kg or 3.5%) could be considered practically very small.

**Figure 3:**
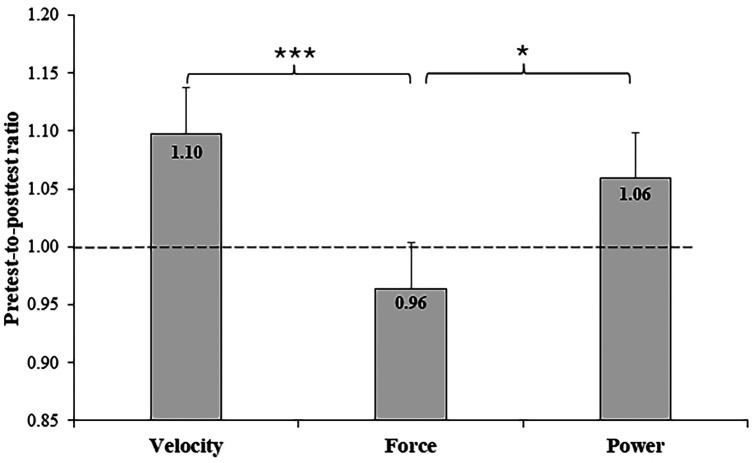
Pre- to post-test ratio of velocity, force, and power. The ratios above 1.0 for velocity and power reflect potentiation effects, whereas the value below 1.0 for force indicates load-type specificity rather than fatigue.

Figure 3 illustrates the pre- to post-test ratios for velocity, force, and power. The analysis of pre- to posttest ratio showed significant improvements in both velocity (*p* < .01) and Pmax (*p* < .05) (i.e., above 1) compared to Fmax ratio. The ratios above 1.0 for velocity and power confirm the potentiation effect induced by inertial loading, while the slight decrease in force (ratio below 1.0) reflects the mechanical specificity of the load rather than fatigue. Moreover, difference of pretest to posttest ratio was very large between velocity and force and large between power and force.

**Figure 4:**
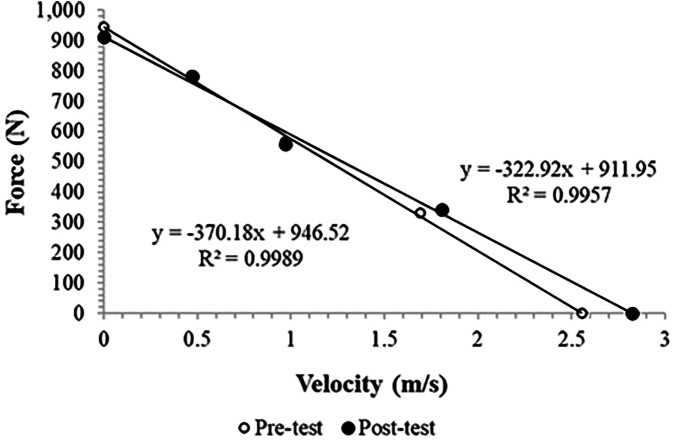
Force–velocity relationships at pre- and post-test. The rightward shift of the regression line denotes enhanced contraction velocity and neuromuscular efficiency following inertial-load–induced potentiation.

The regression analysis of the force–velocity relationship (Figure 4) revealed a rightward shift of the post-test slope, indicating that participants were able to generate higher velocities at comparable force levels. This shift reflects enhanced neuromuscular efficiency and increased rate of force development following inertial-load–induced potentiation. Individual force–velocity regressions demonstrated high linearity in both pre- and post-test conditions (all R^2^ > 0.90). Group-level force–velocity relationships showed a large force-velocity relationship at both pre- and post-test.

## 4 Discussion

In racket sports such as tennis, badminton, table tennis, baseball, and squash stroke performance largely depends on the athlete's ability to generate explosive upper-limb movements under light external loads (22). Enhancing movement velocity and power in these actions is therefore critical for improving shot effectiveness and overall performance. In this context, the present study aimed to investigate the effects of post-activation potentiation (PAP) induced by predominantly inertial loading on velocity, power, force, and the force–velocity relationship. It was hypothesized that PAP with predominatnly inertial loading would enhance velocity and power, have no significant effect on force, and shift the force–velocity relationship to the right. The main findings showed that the utilization of the predominantly inertial load produced PAP effects primarily through increased movement velocity, thereby improving maximal power output. Consequently, the first hypothesis was confirmed. Given the significant decline in Fmax, the second hypothesis was not true, while the observed rightward shift in the force–velocity relationship supported the third.

The improvement in movement velocity following post-activation potentiation (PAP) with predominantly inertial loading can be attributed to enhanced neuromuscular activation and the preferential recruitment of fast-twitch motor units (4). The consistent rightward shift of the F–V curve (Figure 4) supports the notion that inertial loading predominantly enhances velocity-oriented neuromuscular adaptations. The reduced variability observed in post-test performance (Figure 2) suggests that the potentiation effect was generally stable across participants. However, minor inter-individual differences likely reflected variations in the temporal balance between potentiation and fatigue—a well-documented feature of post-activation potentiation responses (23). The 5 min rest interval was selected based on prior evidence showing that moderate recovery durations (5–7 min) optimize upper-body PAP effects in well-trained individuals (7, 24). Considering the moderate volume of the conditioning activity (2 × 3 repetitions at 80%–90% 1RM), this duration was deemed sufficient to minimize fatigue while preserving potentiation. Moreover, inertial loading provides resistance that challenges the neuromuscular system while maintaining high movement velocities, optimizing the balance between force generation and contraction speed. The rapid acceleration and deceleration phases characteristic of inertial loading likely increase motor unit recruitment and firing frequency, particularly in Type II fibers responsible for high-velocity force production (5). In addition, the predominantly inertial nature of the load—with elastic elements compensating gravity—requires continuous modulation of neural drive to control acceleration, which heightens motoneuron excitability and potentiates rate of force development rather than maximal tension. This mechanical context explains why Vmax improved, as faster cross-bridge cycling and enhanced neural activation favor rapid contractions, while Fmax decreased due to the limited time under tension and reduced contribution of slow-twitch, high-force fibers (4, 5, 8). These findings align with previous research demonstrating that PAP protocols emphasizing high-velocity contractions or ballistic movements can improve subsequent explosive performance (8, 12, 25). Thus, inertial loading appears to be an effective stimulus for eliciting PAP-related velocity enhancements, likely due to its ability to optimize neural drive and maximize fast-twitch fiber contribution.

Considering power improvement, our results are in line with the study of Djuric et al. (15) who examined the selective effects of weight-only, weight-plus-inertia, and inertia-only on muscle force, velocity, and power. Over an eight-week training period, participants performed maximal bench press throws with weight-only, weight-plus-inertia, and inertia-only. The results demonstrated that while all loading conditions improved power output, inertia-based training was the most effective, primarily enhancing velocity, whereas weight-only training selectively increased force. Therefore, our findings highlight that the acute effects of inertial loading could be strategically applied to optimize specific neuromuscular adaptations, with inertia-based loading offering a particularly effective method for enhancing explosive performance of upper limbs on account of movement velocity. From a practical perspective, such improvements are directly transferable to racket sports, where stroke performance relies heavily on the ability to generate maximal racket head speed within a short time frame. Enhancing movement velocity through inertial PAP could therefore translate into faster serves, more powerful forehands, and improved shot precision due to optimized neuromuscular readiness.

In contrast, this type of load is not effective or even counterproductive if the goal is to actually improve forceful performance. The observed reduction in Fmax aligns with previous studies demonstrating that inertial loading predominantly enhances contraction velocity while exerting little or no positive effect on maximal force production (13, 15). Such outcomes reflect the inherent mechanical specificity of inertial resistance, where rapid acceleration and short time under tension emphasize rate of force development rather than maximal tension capacity. Furthermore, since previous research with trained individuals confirmed that a 5-minute recovery period is sufficient to minimize fatigue (7), the reduction in Fmax in this study is more likely related to the neuromechanical characteristics of the load than to residual fatigue. Additionally, the decline in Fmax may also reflect a transient shift in neural drive strategy, where preferential activation and partial fatigue of higher-threshold motor units reduce maximal force capacity while sustaining high-velocity output (23). Moreover, Moir et al. (26) investigated the effects of ballistic and non-ballistic bench press exercises, performed at 30% and 90% 1RM, on force, velocity, and power, and found that heavier loads (90% 1RM) resulted in significantly greater forces but lower velocities and power outputs compared to lighter loads (30% 1RM). However, in racket sports, maximal force is not a dominant determinant of success, as performance primarily depends on the ability to generate high movement velocity and precise control of the implement. Therefore, the absence of improvement in force observed in this study should not be viewed as a limitation but rather as an indication that inertial loading specifically targets the velocity–power domain that is crucial for racket sport performance — a region of the force–velocity spectrum where high contraction speeds are coupled with moderate loads to maximize power output (1).

### 4.1 Limitations

This study has several important limitations. Most notably, the inertial load used was not purely inertial. The reference load of 20 kg, comprising the barbell and upper-body segment mass, was not compensated by the elastic bands and therefore retained both gravitational and inertial components. This limitation stemmed from the custom-made Smith machine, which allowed gravitational compensation of up to 60 kg. Although this setup partially compromised the purity of the load—meaning that it was not entirely inertial but predominantly inertia-driven—similar partial compensation approaches have been employed in previous load-type validation studies (13–15). Next, the sample included only young, trained males, limiting generalizability to broader populations. It is of note, however, that this is an advanced training method used typically in competitive athletes. The relatively small sample size, while statistically sufficient, may reduce the ability to detect more nuanced effects. Furthermore, only acute responses were assessed; no long-term effects or transfer to sport-specific tasks were evaluated. The force-velocity profile was derived using just three load conditions, which may reduce the accuracy of the estimated F₀, V₀, and Pmax values; however, this approach represents a conservative compromise during initial testing in acute PAP designs where minimizing fatigue is essential. Future studies should explore PAP responses under different loading types—gravitational, inertial, and combined—as these may differently affect strength, speed, and power. A gravitational load might predominantly enhance force, inertial load may favor velocity, while combined loading could yield balanced improvements, particularly in power, consistent with prior findings.

### 4.2 Practical implications

The findings of this study offer practical insights for strength and conditioning professionals aiming to enhance upper-body explosive performance through post-activation potentiation strategies. Specifically, the use of predominantly inertial loading—applied via a system that neutralizes gravitational resistance—proved effective in acutely increasing movement velocity and power output, while reducing maximal force. This suggests that inertial PAP protocols may be particularly beneficial for athletes in sports that require high-speed upper-limb actions, such as throwing, punching, or stroke movements (e.g., in handball, boxing, or racket sports). However, due to the reduction in force, this method may not be suitable when the primary performance goal is maximal strength. Instead, PAP protocols based on traditional weightlifting may be more appropriate in such cases. Overall, inertial PAP represents a viable and targeted strategy to transiently enhance upper-body power and velocity, particularly when time-efficient and sport-specific activation is required. Specifically, racket sports athletes may benefit from incorporating inertial PAP protocols during warm-up or pre-competition routines to acutely enhance upper-limb power and swing velocity. Given that these sports rely on repetitive, high-speed movements under light resistance, inertial loading provides an ideal modality for improving neuromuscular responsiveness without inducing fatigue.

## 5 Conclusion

In conclusion, the present findings demonstrate that predominantly inertial load-induced post-activation potentiation (PAP) acutely enhances upper-body movement velocity and power while having no significant effect on maximal force output. These outcomes support the use of inertial loading as an effective strategy for eliciting neuromuscular potentiation aimed at improving explosive performance. From a sport-specific perspective, these findings have direct implications for racket sports such as tennis, badminton, table tennis, baseball, and squash where rapid, high-velocity arm movements are essential for generating racket head speed and stroke precision. The ability to transiently enhance movement velocity and power through inertial PAP could contribute to improved serve and stroke performance, particularly when applied as part of warm-up or pre-competition routines. Therefore, incorporating inertial loading protocols may serve as a valuable, time-efficient approach to acutely optimize neuromuscular readiness and enhance technical performance in racket sport athletes.

## Data Availability

The original contributions presented in the study are included in the article/Supplementary Material, further inquiries can be directed to the corresponding author.
